# P-1181. Comparing the outcomes of ceftaroline versus vancomycin for the treatment of osteomyelitis and septic arthritis in pediatric patients

**DOI:** 10.1093/ofid/ofae631.1367

**Published:** 2025-01-29

**Authors:** Patricia Pichilingue Reto, Katherine Spink, Archana Bottu, Ariana Bolumen, Gerienne White, Kelsey Trimble, Rifah Huq, John Vanchiere

**Affiliations:** Louisiana State University Health Sciences Center Shreveport, Shreveport, Louisiana; Louisiana State University Health Sciences Center Shreveport, Shreveport, Louisiana; Louisiana State University Health Sciences Center Shreveport, Shreveport, Louisiana; Louisiana State University Health Sciences Center Shreveport, Shreveport, Louisiana; Louisiana State University Health Sciences Center Shreveport, Shreveport, Louisiana; Ochsner LSU Health Shreveport, Shreveport, Louisiana; Louisiana State University Health Sciences Center Shreveport, Shreveport, Louisiana; Lousiana State University Health Shreveport, shreveport, Louisiana

## Abstract

**Background:**

The most common cause of osteomyelitis and septic arthritis is *Staphylococcus aureus*. Vancomycin is a glycopeptide antibiotic used to treat proven or suspected methicillin-resistant *S. aureus* (MRSA). It is known to have serious adverse effects like nephrotoxicity and vancomycin infusion syndrome. Ceftaroline, a fifth-generation cephalosporin active against MRSA, is not approved by the Food and Drug Administration (FDA) for osteomyelitis or septic arthritis; but it is commonly used to treat bone and joint infections due to its range of activity and safety profile when compared to vancomycin.

**Table 1.**
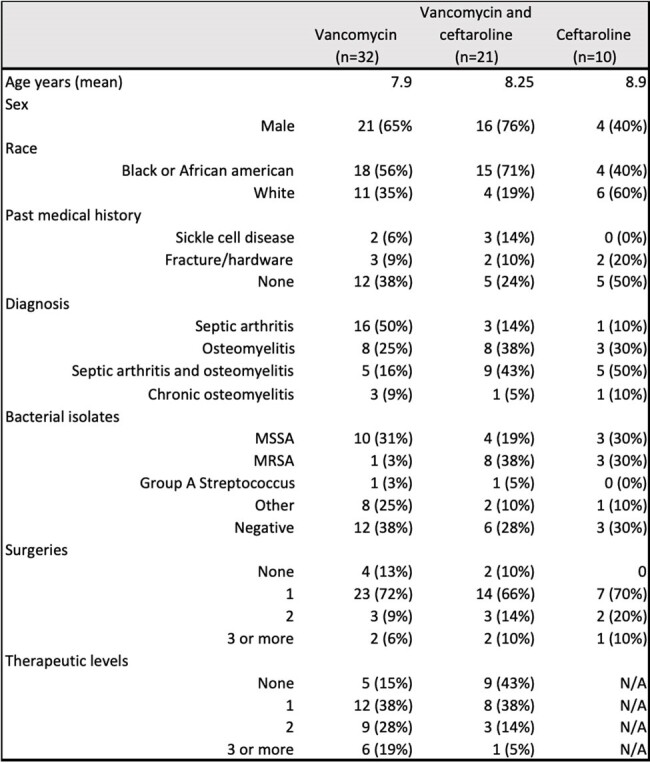
Demographic, clinical and microbiological characteristics of patients treated with vancomycin, vancomycin followed by ceftaroline, and ceftaroline

**Methods:**

This is a retrospective study of osteomyelitis and/or septic arthritis cases in children (0-18 years old) treated with Vancomycin and/or Ceftaroline between October 1^st^, 2019 to October 1^st^, 2023 admitted at Ochsner LSU Health Shreveport. Demographic data, pertinent medical history, days of antibiotic therapy, surgeries and microbiology results were collected. Complications during clinical course and treatment failure were reported.

**Table 2.**
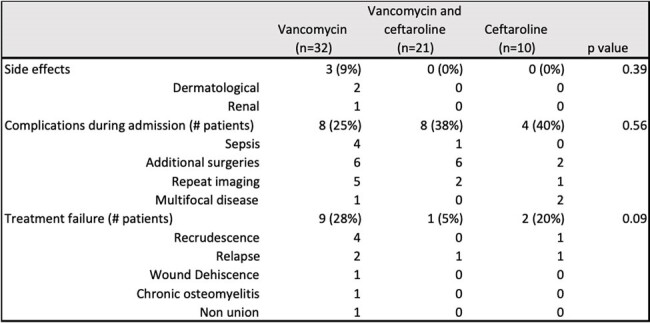
Clinical outcomes of patients treated with vancomycin, vancomycin followed by ceftaroline, and ceftaroline. Fisher’s exact test was performed for all categorical variables.

**Results:**

During the study period, 63 patients were admitted with osteomyelitis and/or septic arthritis and received either vancomycin (n=32), vancomycin followed by ceftaroline (n=21) or ceftaroline (n=10). Demographic, clinical, and microbiological data are presented in Table 1. Among treatment groups, one or more complications were reported per patient during admission. These included sepsis, multifocal disease, repeat imaging and additional surgeries. Treatment failure was defined as recrudescence, relapse, wound dehiscence, chronic osteomyelitis and non-union (Table 2). The vancomycin group had more treatment failures, with 9 patients (28%), when compared to 2 (20%) in the ceftaroline group and 1 (5%) in the group of vancomycin followed by ceftaroline. This difference was not statistically significant (p= 0.09) (Fig. 1). Side effects were only seen in the vancomycin group.

**Figure 1:**
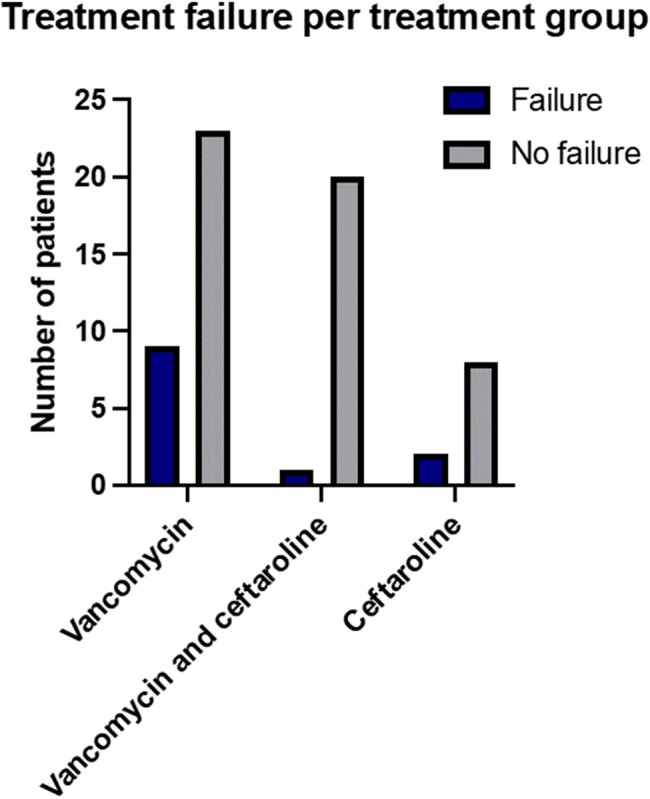
Treatment failure per treatment group.

Treatment failure was reported in 9 (28%) of 32 patients who received vancomycin alone, 2 (20%) of 10 patients on the ceftaroline group and 1 (5%) among 21 patients treated with vancomycin followed by ceftaroline. Fisher’s exact test was performed with a p value of 0.09.

**Conclusion:**

This small retrospective study showed that children treated with vancomycin alone for bone and joint infections experienced more treatment failures. Side effects were only reported with vancomycin alone. Ceftaroline should be considered a safe alternative and promising empiric agent to treat osteomyelitis and septic arthritis in children.

**Disclosures:**

**John Vanchiere, MD, PhD**, Biocryst: Advisor/Consultant|Biocryst: Grant/Research Support|Enanta: Grant/Research Support|GSK: Grant/Research Support|Merck: Grant/Research Support|Pfizer: Grant/Research Support|Tetraphase Pharmaceutical: Advisor/Consultant|Tetraphase Pharmaceutical: Grant/Research Support

